# Loss Aversion Correlates With the Propensity to Deploy Model-Based Control

**DOI:** 10.3389/fnins.2019.00915

**Published:** 2019-09-06

**Authors:** Alec Solway, Terry Lohrenz, P. Read Montague

**Affiliations:** ^1^Virginia Tech Carilion Research Institute, Roanoke, VA, United States; ^2^Department of Physics, Virginia Polytechnic Institute and State University, Blacksburg, VA, United States; ^3^Wellcome Trust Centre for Neuroimaging, University College London, London, United Kingdom

**Keywords:** reinforcement learning, model-based, planning, neuroeconomics, subjective utility, loss aversion

## Abstract

Reward-based decision making is thought to be driven by at least two different types of decision systems: a simple stimulus–response cache-based system which embodies the common-sense notion of “habit,” for which model-free reinforcement learning serves as a computational substrate, and a more deliberate, prospective, model-based planning system. Previous work has shown that loss aversion, a well-studied measure of how much more on average individuals weigh losses relative to gains during decision making, is reduced when participants take all possible decisions and outcomes into account including future ones, relative to when they myopically focus on the current decision. Model-based control offers a putative mechanism for implementing such foresight. Using a well-powered data set (*N* = 117) in which participants completed two different tasks designed to measure each of the two quantities of interest, and four models of choice data for these tasks, we found consistent evidence of a relationship between loss aversion and model-based control but in the direction opposite to that expected based on previous work: loss aversion had a positive relationship with model-based control. We did not find evidence for a relationship between either decision system and risk aversion, a related aspect of subjective utility.

## 1. Introduction

Previous work has shown that thinking about all possible decision contexts rather than just the current one reduces loss aversion (Sokol-Hessner et al., 2009, 2012), a well-studied measure of how much more on average individuals weigh losses relative to gains (Kahneman and Tversky, 1979). In this work, participants performed a standard descriptive decision making task in which they chose between a gamble and a sure outcome presented in numeric form. In one condition, participants were told to myopically pay attention only to the current trial. In another condition, participants were told to think about each trial in the context of all previous and future possible decisions and outcomes. Relative to the myopic condition, loss aversion was reduced when participants were told to treat each decision as one of many.

Two immediate questions stem from this work. The first is whether *baseline* differences in the propensity to consider the entirety of the decision context, without explicit instruction, relate to differences in loss aversion. The second is how this ability may be formally characterized. Model-based control offers one possible mechanism through which potential future decisions may be simulated (Daw et al., 2005, 2011).

Imagine moving to a new town and having to learn the route to the nearest grocery store. Exploring the town, one strategy you can employ is to track how successful each turn was in getting you to your goal. For example, upon arriving at the store, if you made a left turn from the last street that you were on, you would strengthen the value of making a left from that street. Next time through, you might notice that you made a right turn from the street before that, and strengthen its value based on the immediate cost or reward (e.g., traffic traveling down the street) and the reward for the rest of the way (getting to the store). Although this description is somewhat simplified from the algorithm the brain is thought to actually use, it captures in principle one strategy to learning the route: updating the value of each action in each state through trial-and-error. Learning and decision making based on this scheme is uncomplicated, however, it is also inflexible. For example, consider what would happen if after learning the route, one of the streets was closed due to construction. In order to learn which of the previous paths were no longer valid, you would have to bump into the new information from a number of them, and slowly propagate this information backward from the affected state.

A different, but related strategy, would entail learning a model of the environment. You can learn how to navigate between streets independently of any goal, learning for example, that turning right from street A leads to street B. Separately, you can also learn the reward or cost associated with each action, for example, that turning right from street A is more costly than turning left because it leads to more traffic. When it comes time to plan a route, you can integrate these two pieces of information on-line to generate the optimal sequence of actions. This type of operation is more computationally intensive than simply recalling the best sequence of turns, but it allows for tremendous flexibility, and can save time and minimize costs both during initial learning and when the environment changes. These advantages result from the ability to learn each component of the model separately, and to propagate at decision time information about changes in one state to all of the states in the environment, without having to revisit them. Faced with a road closure, the relevant part of the model can be modified, and the transition and reward information re-combined to generate a new route from any starting location.

Laboratory studies suggest the brain employs both classes of strategies, each supported by separate, but not entirely independent, neural hardware (Killcross and Coutureau, 2003; Valentin et al., 2007; Daw et al., 2011). The study of the decision systems that implement these strategies, and debate about their scope, is hardly new (Tolman, 1932; Hull, 1943). More recently, these two systems have been formalized within the framework of reinforcement learning (Daw et al., 2005, 2011). The less complex system is habit-like, learning stimulus–response associations retrospectively from experience using “model-free” reinforcement learning algorithms. It is fast and reflexive, but relatively unsophisticated, and sometimes prone to error. The more complex system is more flexible and accurate, learning and combining separate information about the environment's transition and reward structure to generate novel prospective plans using “model-based” reinforcement learning algorithms. The last several years have seen an explosion in work using this framework both to study how these systems operate, and how a variety of individual differences map on to the balance between them in individuals (e.g., Daw et al., 2005, 2011; Glascher et al., 2010; Keramati et al., 2011; Simon and Daw, 2011; Huys et al., 2012; Solway and Botvinick, 2012, 2015; Otto et al., 2013, 2014; Lee et al., 2014; Doll et al., 2015a,b; Voon et al., 2015).

In the present work, we asked whether the propensity for model-based control and the balance between the two decision systems was related to loss aversion. We hypothesized that model-based control may be a computational substrate for the forward simulations involved in thinking through possible decisions and outcomes, and since this has been shown to reduce loss aversion (Sokol-Hessner et al., 2009, 2012), increased model-based control would be correlated with reduced loss aversion.

Participants (*N* = 117) completed two different tasks, each previously designed and used to measure one of the dimensions of interest. Analyzing the data using four different models, we found consistent evidence of a relationship between loss aversion and model-based control, but in the opposite direction to what would be expected based on previous work. Individuals that employed more model-based control were more loss averse. We also controlled and tested for a relationship between model-based and model-free control and risk aversion, but did not find evidence of a relationship with either decision system under any model formulation.

## 2. Methods

### 2.1. Participants

One hundred and seventeen participants completed both experiments as part of the Roanoke Brain Study, a large scale study on individual differences. This study was carried out in accordance with the recommendations of the Institutional Review Board at Virginia Tech with written informed consent from all subjects. All subjects gave written informed consent in accordance with the Declaration of Helsinki. The protocol was approved by the Institutional Review Board at Virginia Tech. All participants were included in the analysis.

### 2.2. Two-Step Task

**Figure 1:**
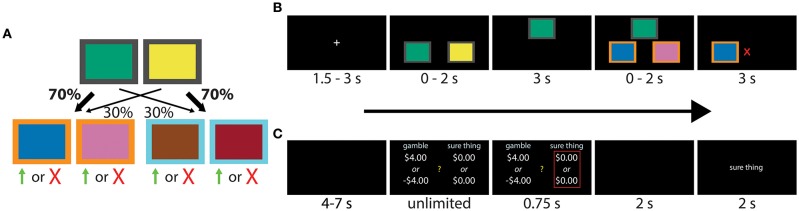
**(A,B)** Transition structure and sequence of events during each experimental trial of the two-step task. Participants had to make a decision between two fractal images, each leading probabilistically to another pair of images and a second decision. The four second-stage images were associated with independently varying binary payoff probabilities, with reward signaled by a green up arrow (point) or red “X” (no point). The task can be solved using either a model-free (“habitual”) or model-based (“goal-directed”) strategy. Images of fractals were used in the actual experiment, here replaced by colored boxes for publication. Modified from Solway et al. (2017). **(C)** Sequence of events within each trial of the gambling task. Participants were asked to make a series binary decisions between a 50/50 gamble and a “sure amount” of money.

The task used to assess differences in model-free and model-based control has previously been reported in a number of studies (e.g., Daw et al., 2011; Otto et al., 2013, 2014; Voon et al., 2015), including by our own group (Solway et al., 2017). Each trial began with a fixation cross, followed by a choice between two fractal images positioned in a horizontal orientation in random order. One image predominantly led (with 70% probability) to a second pair of images, but sometimes to a third pair (with 30% probability). The second first-stage image had the reverse mapping, predominantly leading to the third pair of images (also with 70% probability), but sometimes to the second pair (with 30% probability; Figure 1A). Participants were informed of the rules, but not which first-stage image led to which pair of second-stage images. This mapping had to be learned through trial-and-error. After participants made the first decision, the chosen image moved to the top of the screen, and the second image disappeared, confirming their selection. Following a short delay, the second set of images appeared according to the rules above. Figure 1B provides a visual depiction of the events within a single trial.

Each second-stage image was associated with a binary payoff whose probability followed an independent Gaussian drift with mean 0 and standard deviation 0.025, and reflecting boundaries at 0.2 and 0.8. Different randomly drifting payoff probabilities were used for each participant. Successful trials were awarded a point, signaled by a green up arrow, and unsuccessful trials ended with no change in points, signaled by a red “X.” The points earned were converted to a monetary bonus ($0.10 per point). Each decision stage had a two second deadline. If the deadline was missed, the text “TOO LATE, NO MONEY EARNED” appeared and the trial was aborted. Partial trials resulting from missed deadlines were excluded in the analysis. Each participant completed 201 trials.

### 2.3. Gambling Task

We used a standard gambling task (Tom et al., 2007; Sokol-Hessner et al., 2012), shown in Figure 1C, to measure loss aversion and risk preferences. On each trial, participants selected between a “sure amount” and a gamble. Selecting the sure amount guaranteed that amount of money, while the gamble was associated with two outcomes, each with a 50% chance of occurring.

There were two types of trials. On “mixed valence” trials, one of the gamble outcomes was a gain, and the other was a loss. The sure amount was always zero. On “gain only” trials, one of the gamble outcomes was a gain, and the other was zero. The sure amount was a smaller gain. Each participant played 60 mixed valence and 20 gain only trials in random order. For mixed valence trials, gains were {$2, $4, $5, $6, $8, $9, $10, $12}, and losses were multiples of -0.25 to -2, in increments of 0.125, of the gain amounts (Sokol-Hessner et al., 2012). Offer values for gain only trials ranged $2–$30 for gambles and $1–$13 for the sure thing.

Each trial began with a fixation cross, followed by the competing offers displayed on different sides of the screen (Figure 1C). The side of the gamble and the sure amount was chosen at random. Choices were confirmed with a red outline, which was followed by the outcome. If participants selected the sure amount, the screen said “no gamble.” Otherwise, the computer played the gamble and reported “win” or “lose.” Participants started with $30 and were told that a random trial would be selected at the end of the experiment whose outcome would be added to or subtracted from this amount.

### 2.4. Two-Step Regression Analysis

The logistic regression and reinforcement learning analyses of the two-step task followed previous work (Daw et al., 2011; Otto et al., 2013, 2014; Solway et al., 2017), including our own. For completeness, we repeat and make explicit here the details within the current context.

(1) $$stay\;\sim Bernoulli\left(\frac{1}{1+\exp(-x)}\right),$$

$$\begin{array}{l} x=\beta_{stay}+\\ \;\beta_{reward}\cdot \;reward+\\ \;\beta_{common}\cdot \;common+\\ \;\beta_{reward\times common}\cdot \;reward\;\times \;common+\\ \;\beta_{loss}\cdot \;loss+\\ \;\beta_{risk}\cdot \;risk+\\ \;\beta_{temperature}\cdot \;temperature+\\ \;\beta_{reward\times loss}\cdot \;reward\times loss+\\ \;\beta_{reward\times risk}\cdot \;reward\times \;risk+\\ \;\beta_{reward\times temperature}\cdot \;reward\;\times \;temperature+\\ \;\beta_{common\times loss}\cdot \;common\times loss+\\ \;\beta_{common\times risk}\cdot \;common\;\times \;risk+\\ \;\beta_{common\times temperature}\cdot \;common\;\times \;temperature+\\ \;\beta_{reward\times common\times loss}\cdot \;reward\times common\;\times \;loss+\\ \;\beta_{reward\times common\times risk}\cdot \;reward\times common\times risk+\\ \;\beta_{reward\times common\times temperature}\cdot \;reward\times \text{ ​}common\;\times \text{​ }temperature. \end{array}$$

The variable *stay* took on value 1 or 0 depending on whether or not the same first-stage action (fractal image) was chosen on the previous trial. *reward* took on value 1 or −1 depending on whether the previous trial was rewarded, and *common* took on value 1 or −1 depending on whether the transition between the first and second stage on the last trial was common or rare. *loss* is the z-scored log loss aversion estimate, *risk* is the z-scored risk preference estimate, and *temperature* is the z-scored inverse softmax temperature from the gambling task, all described below. As described in more detail in *Results*, the main effect of reward is a proxy for model-free control, the interaction between reward and transition type is a proxy for model-based control, and the interaction of each with a gambling task variable determines the extent to which that variable scales with the respective system.

The regressions were performed using a hierarchical Bayesian formulation. β_*stay*_, β_*reward*_, β_*common*_, and β_*reward*×*common*_ were instantiated once per participant, each drawn from a group level Gaussian with a relatively broad *N*(0, 2^2^) prior on the mean and a half-Cauchy(0, 2.5) prior on the standard deviation. The remaining regression coefficients were instantiated once at the group level with a *N*(0, 2^2^) prior.

### 2.5. Two-Step Reinforcement Learning Model

The model-free component learned a table of action values, *Q*(*s, a*). The environment consisted of three primary states, one for the first-stage decision, and one for each possible second-stage decision, and two actions in each state, corresponding to the fractal images. Q-values were initialized to 0.5 (mid-way between the two known extreme values) and updated according to SARSA(λ) (Rummery and Niranjan, 1994):

(2) $$\begin{array}{l} Q_{mf}(s_{t,i},a_{t,i})=Q_{mf}(s_{t,i},a_{t,i})+\alpha (r_{t,i}+Q_{mf}(s_{t,i+1},a_{t,i+1})\\ \;-Q_{mf}(s_{t,i},a_{t,i})). \end{array}$$

*t* refers to the trial number and *i* to the decision stage. *r*_*t, i*_ is the immediate reward, always 0 following the first stage, and 1 or 0 following the second stage. *Q*_*mf*_(*s*_*t*, 3_, *a*_*t*, 3_) was set to 0 because there was no third stage. An eligibility trace updated first-stage Q-values according to the second-stage outcome:

(3) $$\begin{array}{r} Q_{mf}\left(s_{t,1},a_{t,1}\right)=Q_{mf}\left(s_{t,1},a_{t,1}\right)+\alpha \lambda \left(r_{t,2}-Q_{mf}\left(s_{t,2},a_{t,2}\right)\right). \end{array}$$

Traces were reset at the beginning of each trial. We found λ to be difficult to identify when allowing it to vary as a free parameter. For simplicity, it was fixed to 1.

Non-chosen action values decayed to baseline:

(4) $$\begin{array}{r} Q_{mf}\left(s,a\right)=Q_{mf}\left(s,a\right)+\alpha \left(0.5-Q_{mf}\left(s,a\right)\right). \end{array}$$

At the second stage, the model-based controller used the same temporal-difference learning rule, and *Q*_*mb*_(*s*_*t*, 2_, *a*_*t*, 2_) = *Q*_*mf*_(*s*_*t*, 2_, *a*_*t*, 2_). Following previous work, the transition function used the veridical values (0.7 and 0.3), and the mapping of the first-stage action to the predominant second-stage state was assigned based on the difference between the number of times the first action led to the first second-stage pair plus the second action led to the second second-stage pair, and the number of times the opposite transitions were observed. A single backup operation using the Bellman equation was used to combine the reward and transition functions and compute model-based action values at the first stage:

(5) $$\begin{array}{r} Q_{mb}\left(s_{t,1},a_{t,1}\right)=\underset{s^{\prime }=\left\{2,3\right\}}{\sum }p\left(s^{\prime }|s_{t,1},a_{t,1}\right)\underset{a=\left\{1,2\right\}}{\max}Q_{mb}\left(s^{\prime },a\right). \end{array}$$

Action selection was conducted using a softmax choice rule. At stage one:

(6) $$\begin{array}{l} p(a|s)=\\ \frac{\exp(\beta_{mb}Q_{mb}(s,a)\;+\;\beta_{mf}Q_{mf}(s,a)\;+\;p\;\cdot \;rep(a)\;+\;\beta_{bias}\cdot \;bias(a))}{\sum_{a^{\prime }}\exp(\beta_{mb}Q_{mb}(s,a^{\prime })\;+\;\beta_{mf}Q_{mf}(s,a^{\prime })\;+\;p\;\cdot \;rep(a^{\prime })\;+\;\beta_{bias}\cdot \;bias(a^{\prime }))}. \end{array}$$

The function *rep*(*a*) was 1 when *a* was the action taken during the first stage of the previous trial, and 0 otherwise. *p* captures the tendency to repeat (*p* > 0) or switch (*p* < 0) actions irrespective of value. The function *bias*(*a*) was 1 for the second action (arbitrarily chosen) and 0 for the first action. This incorporates bias toward the first action when β_*bias*_ is negative.

At the second stage, action selection was dependent on a single set of *Q*-values:

(7) $$\begin{array}{l} p(a|s)=\frac{\exp(\beta_{2}Q_{mf}(s,a))}{\sum_{a^{\prime }}\exp(\beta_{2}Q_{mf}(s,a^{\prime }))}. \end{array}$$

There were six parameters in all: α, β_*mb*_, β_*mf*_, β_2_, *p*, and β_*bias*_. Each parameter was instantiated separately for each participant. Subject level parameters were modeled as being drawn from a group level Gaussian similar to the regression model above. An exception to this are the bias parameters, which captured individual nuance and had independent Gaussian priors. Parameters governing the strength of model-based and model-free control also incorporated the possible effects of the gambling task parameters:

(8) $$\begin{array}{l} \beta_{mb}\sim N(\beta_{mb}^{\mu }+\beta_{mb,loss}\cdot \;z\text{-}score(log(loss))+\\ \;\beta_{mb,risk}\cdot \;z\text{-}score(risk)+\beta_{mb,temperature}\\ \;\cdot \;z\text{-}score(temperature),\beta_{mb}^{\sigma }) \end{array}$$

and similarly for β_*mf*_. The learning rate, α, was transformed to the (0, 1) range using the logistic function before being applied. The hyperprior on each group level mean was a broad *N*(0, 10^2^) Gaussian (with the exception of the group learning rate, which had a *N*(0, 5^2^) prior), with a half-Cauchy(0, 2.5) for the standard deviation.

### 2.6. Gambling Task Model

We modeled the gambling task separately with two types of utility functions. The first was based on prospect theory (Tversky and Kahneman, 1992):

(9) $$\begin{array}{l} U_{1}(v)=\{\begin{array}{ll} v^{\gamma } & \text{if }v\geq 0,\\ -\kappa {\left(-v\right)}^{\gamma } & \text{otherwise}. \end{array} \end{array}$$

For simplicity, because the task used a single set of probabilities, we assumed they took on their veridical values without special weighting (Sokol-Hessner et al., 2009). The parameter κ is loss aversion (the ratio of the weight on losses relative to the weight on gains), and 1-γ is a measure of risk aversion. Although prospect theory allows γ to take on separate values for gains and losses, previous work with this task has constrained models to use a single parameter for both (Sokol-Hessner et al., 2009, 2012, 2015a,b) because there is a tradeoff with κ: a preference against gambles on mixed valence trials can result either by setting κ or a loss specific γ to be high. We follow this approach for modeling mixed valence trials. However, if the true data generating process has separate γ parameters for gains and losses, estimates obtained in this way may be biased, representing a mixture of the two underlying values. This would bias our analysis of risk preferences. To get around this, we modeled gain only trials with a separate γ parameter, and used this parameter as the measure of risk preference on which we focus.

Action selection was again modeled using a softmax choice rule. For a gamble with outcomes *g*_1_ and *g*_2_, and sure amount *s*,

(10) $$\begin{array}{r} p\left(gamble\right)=\frac{\exp\left(\theta \left(0.5\;\cdot \;U_{1}\left(g_{1}\right)\;+\;0.5\;\cdot \;U_{1}\left(g_{2}\right)\right)\right)}{\exp\left(\theta \left(0.5\;\cdot \;U_{1}\left(g_{1}\right)\;+\;0.5\;\cdot \;U_{1}\left(g_{2}\right)\right)\right)\;+\;\exp\left(\theta U_{1}\left(s\right)\right)}. \end{array}$$

The model has four parameters in all: κ, γ_*m*_ (for mixed valence trials), γ_*g*_ (for gain only trials, with 1 − γ_*g*_ the measure of risk aversion in the main text), and θ, instantiated once for each participant.

The second function we tested assumed that utilities are linear in value, but that there is also a penalty linear in the standard deviation of the gamble:

(11) $$U_{2}(v)\;=\{\begin{array}{ll} v & \text{if }v\geq 0\\ \kappa v & \text{otherwise}, \end{array}$$

(12) $$\begin{array}{r} U_{3}\left(g_{1},g_{2}\right)=0.5\cdot U_{2}\left(g_{1}\right)+0.5\cdot U_{2}\left(g_{2}\right)-w\sigma , \end{array}$$

where σ is the standard deviation of outcomes *U*_2_(*g*_1_) and *U*_2_(*g*_2_). This model has three parameters: κ, *w*, and θ, instantiated once for each participant.

The parameters γ, *w*, and θ were modeled as being drawn from a group level half-Gaussian defined on [0, ∞). κ was drawn from a log-normal distribution (Sokol-Hessner et al., 2012, 2015a,b). Hyperpriors were *N*(0, 2^2^) for the mean of κ, γ, and *w*, *N*(0, 10^2^) for the mean of θ, and half-Cauchy(0, 2.5) for each standard deviation.

### 2.7. Model Fitting

Model fitting procedures were similar to previous work (Solway et al., 2017). Inference for each combination of models was performed via Markov chain Monte Carlo, using the No-U-Turn sampler (Hoffman and Gelman, 2014) implemented in Stan (Stan Development Team). Proper mixing was assessed by ensuring the $\hat{R}$ statistic was less than 1.1 for all variables (Gelman and Rubin, 1992), and qualitatively by eye. Eight chains were run in parallel for 4,000 samples (10,000 for the regression models), using the first 1,000 for warmup. The posterior was estimated with the resulting 24,000 samples (72,000 for the regression models). We fit each of four combinations of models simultaneously to data for all subjects and both experiments: each version of the two-step model (the logistic regression and hybrid reinforcement learning model) crossed with each utility function for the gambling task.

## 3. Results

To test whether individuals scale model-based control with loss aversion, participants completed two well-studied tasks, each designed to separately measure one of the two dimensions of interest. The first task (Daw et al., 2011; Otto et al., 2013, 2014; Solway et al., 2017), designed to measure decision system control, required two decisions to be made on each trial (Figures 1A,B). The first-stage decision was always between the same two actions, represented by fractal images displayed on a computer screen. One action predominantly led to a second pair of images, but a portion of the time transitioned to a third pair. The second first-stage action had the reverse mapping, predominantly leading to the third pair of images, but sometimes to the second pair (see Figure 1A). Participants had to then make a second decision between the new pair of images that appeared, which resulted in a probabilistic payoff. Payoff probabilities drifted randomly and independently for each of the four second-stage images, requiring participants to balance exploration and exploitation. To measure loss aversion, participants completed a standard gambling task (Tom et al., 2007; Sokol-Hessner et al., 2012) where on each trial they chose between a sure amount of money and a 50/50 gamble (Figure 1C). The two experiments were performed in separate sessions of the Roanoke Brain Study, a large scale study of individual differences (time between sessions ranged 1–674 days).

There are two established ways of estimating model-free and model-based control in the first task, and two popular utility functions that are used when modeling the second task. We examined the relationships of interest separately under all 2 × 2 models.

The first measure of decision system control in the two-step task results from comparing the first-stage choice on consecutive trials and considering whether participants made the same decision as a function of: (1) whether the last trial was rewarded, and (2) whether the state transition from the first stage to the second stage was common or rare (Daw et al., 2011; Otto et al., 2013, 2014; Solway et al., 2017). Because a model-free controller does not take the transition structure into account, and learns retrospectively from reward, it predicts higher stay probabilities for rewarded compared to unrewarded trials regardless of transition type. A model-based controller, which has access to the transition structure, predicts higher stay probabilities for rewarded trials following common transitions, and unrewarded trials following rare transitions (correctly attributing the lack of reward to the less common transition), and lower stay probabilities for the two opposite situations. Rather than consider only two trials at a time, a second measure of decision system control can be obtained by fitting a hybrid reinforcement learning model to each participant's full decision history (Daw et al., 2011; Otto et al., 2013; Solway et al., 2017).

For decisions made in the gambling task, it is standard practice to assume that veridical outcome values are transformed using a subjective utility function, but the form of this function is debated (d'Acremont and Bossaerts, 2008). We consider two widely used variants. The first, a version of prospect theory, assumes that there is a differential weight on losses, and that values are transformed using a power function (Tversky and Kahneman, 1992; Sokol-Hessner et al., 2012). The latter feature results in risk averse, or risk seeking behavior, a point we return to below. A second common form of the subjective utility function (the “mean–variance” approach) assumes that individual values are linear, but that there is a penalty for gambles proportional to their variance or standard deviation.

For each of the four models considered, we used a hierarchical modeling approach to simultaneously fit the data for all subjects from both experiments, and estimate the relationship between variables across tasks. For details, see section Methods. Rather than focusing on idiosyncratic results from a single model, we looked for a pattern of consistent results across all four models. Our initial analysis focused on looking at pairs of trials in the two-step task, and using the prospect theory utility function for outcomes in the gambling task. We performed a logistic regression predicting first-stage stay probabilities from reward, transition type (common or rare), the interaction of reward and transition type, and the interaction of each with loss aversion. As described above, the main effect of reward is a proxy for the strength of model-free control, and the interaction between reward and transition type is a proxy for the strength of model-based control. Both terms were significant, implying participants used both strategies in the task (see Table 1 for the main parameters of interest and Table S1 for auxiliary parameter estimates). The interaction of each term with loss aversion determines whether the respective system scales with loss aversion. The three-way interaction between reward, transition type, and loss aversion was significant and positive, whereas the interaction between reward and loss aversion was not significant.

**Table 1:** Group level estimates of model-based and model-free control in the two-step task, and the effects of the gambling task variables.

**Parameter**	**Description**	**Median & 95% credible interval**	**1-p(x>0)**
*Reward*	Model-free control	0.350 (0.267, 0.433)	0.000
*Reward* × *loss*	Effect of loss aversion on model-free control	0.040 (−0.049, 0.131)	0.190
*Reward* × *risk*	Effect of risk aversion on model-free control	−0.051 (−0.145, 0.043)	0.857
*Reward* × θ	Effect of gamble inverse temperature on model-free control	0.073 (−0.063, 0.216)	0.149
*Reward* × *common transition*	Model-based control	0.139 (0.083, 0.195)	0.000
*Reward* × *common* × *loss*	Effect of loss aversion on model-based control	0.067 (0.006, 0.128)	0.017
*Reward* × *common* × *risk*	Effect of risk aversion on model-based control	−0.011 (−0.075, 0.053)	0.631
*Reward* × *common* × θ	Effect of gamble inverse temperature on model-based control	0.105 (0.010, 0.195)	0.015
*Reward* × *common* × *loss*− *reward* × *loss*	Differential effect of loss aversion on model-based control	0.026 (−0.087, 0.140)	0.325
*Reward* × *common* × *risk*− *reward* × *risk*	Differential effect of risk aversion on model-based control	0.041 (−0.076, 0.158)	0.248
*Reward* × *common* × θ− *reward* × θ	Differential effect of gamble inverse temperature on model-based control	0.031 (−0.133, 0.187)	0.351

*Based on the logistic regression/prospect theory analysis*.

**Figure 2:**
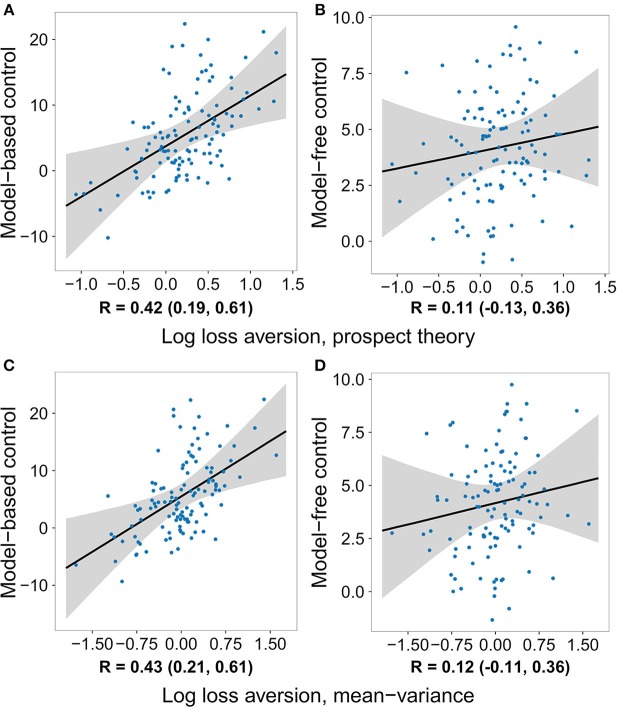
The degree to which individuals deploy each decision system versus the logarithm of their loss aversion weight, after removing the effects of risk aversion and the gambling task inverse softmax temperature. Control estimates are based on a hybrid reinforcement learning model fit to the two-step data, while loss aversion was estimated using two different utility functions applied to the gambling task data. Each dot represents the medians of one subject's parameter estimates, the black line is the median regression line, and the gray area outlines the 95% credible interval. Below each panel is a summary of the posterior distribution of the respective correlation coefficient. The posterior is a more appropriately conservative summary of the relationship between the two quantities than the correlation between the median values, because it takes into account the uncertainty in each subject's parameter estimates. A relationship between loss aversion and model-based control can be seen using both utility functions, while a relationship with model-free control is not seen with either one. **(A)** Model-based control vs. log loss aversion measured using the prospect theory model. **(B)** Model-free control vs. log loss aversion measured using the prospect theory model. **(C)** Model-based control vs. log loss aversion measured using the mean-variance model. **(D)** Model-free control vs. log loss aversion measured using the mean-variance model.

The same pattern of effects was observed using control estimates based on a hybrid reinforcement learning model fit to the participants' full decision history (Table 2 and Table S2). The model used closely follows previous work (Daw et al., 2011; Otto et al., 2013; Solway et al., 2017) (for details see section Methods). The top row of Figure 2 plots the relationship between the strength of each decision system estimated in this way and loss aversion estimated using the prospect theory utility function. Critically, not only was the effect on model-based control significant and the effect on model-free control not significant, but their difference was significant (Figure S1). Repeating the same logistic regression and reinforcement learning based analyses using a mean–variance utility function to model the gambling data revealed the same pattern of effects (see Tables 3, 4, Tables S3, S4, and the bottom row of Figure 2).

**Table 2:** Group level estimates of model-based and model-free control in the two-step task, and the effects of the gambling task variables.

**Parameter**	**Description**	**Median & 95% credible interval**	**1-p(x>0)**
β_*mf*_	Model-free control	4.189 (3.384, 5.108)	0.000
β_*mf, loss*_	Effect of loss aversion on model-free control	0.360 (−0.657, 1.316)	0.237
β_*mf, risk*_	Effect of risk aversion on model-free control	−0.610 (−1.591, 0.324)	0.904
β_*mf*, θ_	Effect of gamble inverse temperature on model-free control	0.057 (−1.116, 1.272)	0.463
β_*mb*_	Model-based control	5.464 (3.375, 7.727)	0.000
β_*mb, loss*_	Effect of loss aversion on model-based control	3.608 (1.083, 6.242)	0.002
β_*mb, risk*_	Effect of risk aversion on model-based control	−1.584 (−4.067, 0.929)	0.894
β_*mb*, θ_	Effect of gamble inverse temperature on model-based control	1.400 (-1.933, 4.720)	0.206
β_*mb, loss*_ − β_*mf, loss*_	Differential effect of loss aversion on model-based control	3.260 (0.468, 6.163)	0.011
β_*mb, risk*_ − β_*mf, risk*_	Differential effect of risk aversion on model-based control	−0.964 (−3.705, 1.780)	0.756
β_*mb*, θ_ − β_*mf*, θ_	Differential effect of gamble inverse temperature on model-based control	1.339 (−2.176, 4.799)	0.228

*Based on the hybrid reinforcement learning model/prospect theory analysis*.

**Table 3:** Group level estimates of model-based and model-free control in the two-step task, and the effects of the gambling task variables.

**Parameter**	**Description**	**Median & 95% credible interval**	**1-p(x>0)**
*Reward*	Model-free control	0.347 (0.263, 0.432)	0.000
*Reward* × *loss*	Effect of loss aversion on model-free control	0.048 (−0.045, 0.140)	0.152
*Reward* × *risk*	Effect of risk aversion on model-free control	−0.040 (−0.143, 0.063)	0.777
*Reward* × θ	Effect of gamble inverse temperature on model-free control	0.048 (−0.054, 0.148)	0.177
*Reward* × *common transition*	Model-based control	0.135 (0.079, 0.192)	0.000
*Reward* × *common* × *loss*	Effect of loss aversion on model-based control	0.074 (0.012, 0.136)	0.010
*Reward* × *common* × *risk*	Effect of risk aversion on model-based control	0.030 (−0.037, 0.098)	0.186
*Reward* × *common* × θ	Effect of gamble inverse temperature on model-based control	0.089 (0.015, 0.164)	0.010
*Reward* × *common* × *loss*− *reward* × *loss*	Differential effect of loss aversion on model-based control	0.026 (−0.089, 0.143)	0.327
*Reward* × *common* × *risk*− *reward* × *risk*	Differential effect of risk aversion on model-based control	0.070 (−0.057, 0.197)	0.138
*Reward* × *common* × θ− *reward* × θ	Differential effect of gamble inverse temperature on model-based control	0.041 (−0.087, 0.171)	0.267

*Based on the logistic regression/mean–variance analysis*.

**Table 4:** Group level estimates of model-based and model-free control in the two-step task, and the effects of the gambling task variables.

**Parameter**	**Description**	**Median & 95% credible interval**	**1-p(x>0)**
β_*mf*_	Model-free control	4.175 (3.385, 5.082)	0.000
β_*mf, loss*_	Effect of loss aversion on model-free control	0.394 (−0.614, 1.328)	0.212
β_*mf, risk*_	Effect of risk aversion on model-free control	−0.431 (−1.428, 0.549)	0.805
β_*mf*, θ_	Effect of gamble inverse temperature on model-free control	0.263 (−0.688, 1.234)	0.291
β_*mb*_	Model-based control	5.521 (3.403, 7.739)	0.000
β_*mb, loss*_	Effect of loss aversion on model-based control	3.793 (1.282, 6.417)	0.002
β_*mb, risk*_	Effect of risk aversion on model-based control	0.366 (−2.204, 2.986)	0.389
β_*mb*, θ_	Effect of gamble inverse temperature on model-based control	1.992 (−0.681, 4.551)	0.071
β_*mb, loss*_ − β_*mf, loss*_	Differential effect of loss aversion on model-based control	3.407 (0.670, 6.268)	0.008
β_*mb, risk*_ − β_*mf, risk*_	Differential effect of risk aversion on model-based control	0.801 (−1.976, 3.682)	0.288
β_*mb*, θ_ − β_*mf*, θ_	Differential effect of gamble inverse temperature on model-based control	1.721 (−1.151, 4.532)	0.121

*Based on the hybrid reinforcement learning model/mean–variance analysis*.

**Figure 3:**
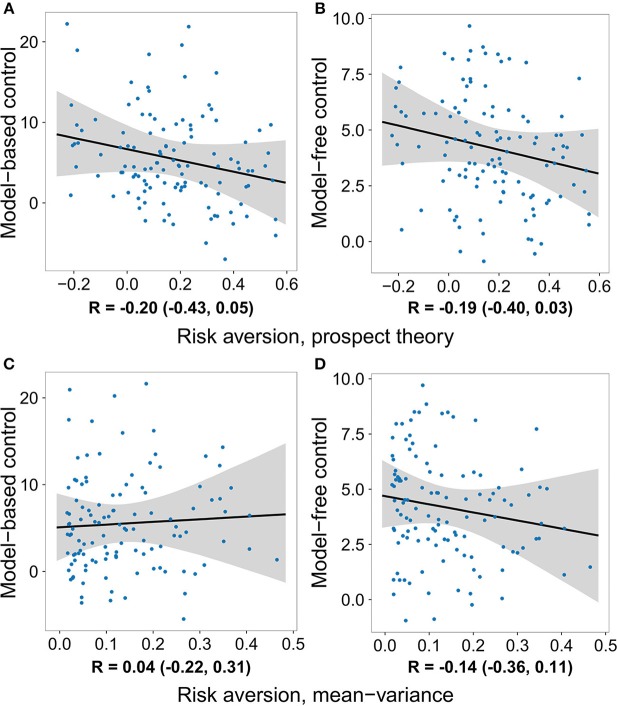
The degree to which individuals deploy each decision system plotted against their degree of risk aversion, after removing the effects of loss aversion and the gambling task inverse softmax temperature. No significant relationship was observed between risk preference and either decision system. **(A)** Model-based control vs. risk aversion measured using the prospect theory model. **(B)** Model-free control vs. risk aversion measured using the prospect theory model. **(C)** Model-based control vs. risk aversion measured using the mean-variance model. **(D)** Model-free control vs. risk aversion measured using the mean-variance model.

Although not within the primary purview of the present work, the gambling data allowed us to also examine the relationship between each decision system and risk preferences. For the prospect theory formulation, we measured risk preference in terms of the concavity of the utility function (Pratt, 1964; Tversky and Kahneman, 1992). In the mean–variance approach, risk aversion is built-in and results from a penalty on a gamble's variance. We tested the relationship between model-based and model-free control and risk preference using all four model formulations, none of which yielded evidence of a significant effect (Tables 1–4 and Figure 3).

The effects of loss and risk aversion on each decision system were tested simultaneously in each of the four formulations. In addition, each analysis also included the inverse softmax temperature parameter from the gambling task as a confound regressor (Tables 1–4 and Figure S2). This parameter can trade off with both loss and risk aversion, and can be interpreted either as a measurement of the degree to which participants engage with the gambling task, or as the independent weight on gain outcomes (loss aversion is the ratio of the weight on loss outcomes to the weight on gain outcomes). We observed a positive relationship between it and model-based control in the logistic regression analyses, but not in the hybrid reinforcement learning model analyses. Notably, the effect of loss aversion on model-based control was observed under all four formulations even when controlling for risk aversion and the inverse softmax temperature.

We also performed two additional confirmatory analyses. To test whether individual performance had an impact on the results, we excluded participants whose 95% credible intervals for performance at both steps (both model-based and model-free control at step 1, and general decision making at step 2) included 0, and re-ran both reinforcement learning model analyses. The results were unchanged: the relationship between model-based control and loss aversion was significant, the relationship between model-free control and loss aversion was not, and the difference was significant. As before, there was no relationship between either decision system and risk aversion or the gambling task inverse temperature. To test the sensitivity of the results to the priors, we multiplied the standard deviations for the priors on all mean effects by 10 and reran both reinforcement learning model analyses. The results under both models were again unchanged.

## 4. Discussion

Previous work has shown that loss aversion, the average weight individuals assign to potential loss relative to gain outcomes during decision making, is reduced when participants take all possible decisions and outcomes into account including future decisions and outcomes, relative to myopically focusing on the present (Sokol-Hessner et al., 2009, 2012). Model-based control offers a putative mechanism for implementing such foresight. Consistent with this idea, focusing on all possible outcomes compared to focusing only on the current decision results in increased activity in the dorsolateral prefrontal cortex (Sokol-Hessner et al., 2012), an area which has been causally linked to model-based control (Smittenaar et al., 2013). We thus hypothesized that increased model-based control would be associated with decreased loss aversion.

We tested this hypothesis using data from two tasks, each designed to measure one of the two quantities of interest, in conjunction with two common ways of modeling each task (four models in all). Contrary to our hypothesis, we found that increased model-based control was associated with *increased* loss aversion. We also tested for but did not observe a relationship between model-based or model-free control and risk aversion.

An explanation for this finding, at present, is lacking. While possible, it is not our contention that the general premise of Sokol-Hessner et al. (2009) and Sokol-Hessner et al. (2012) is incorrect. Instead, counter to intuition, model-based control may not serve as a computational substrate for the prospective activity required to decrease loss aversion. Moreover, a missing mediator or latent factor is likely necessary to explain our particular results, although it is not currently clear what it would be. An appealing possibility is that individual differences in *worry* may link these two quantities. Worriers are apt at running future oriented simulations: when asked to simulate sequences of hypothetical catastrophic outcomes, worriers generate many more steps, reminiscent of simulating forward the transition function in model-based reinforcement learning (Vasey and Borkovec, 1992). Worriers also have an attentional bias toward threat (Bar-Haim et al., 2007; Cisler and Koster, 2010), and overweigh the costs associated with negative outcomes (Butler and Mathews, 1983; Berenbaum et al., 2007a,b). However, recent work has shown that loss aversion specifically is not affected in patients with generalized anxiety disorder (Charpentier et al., 2017), in which worry plays a central role, making this explanation less likely.

Taking the result at face value, there are several additional points of note. First, the two tasks were administered on separate days, for some subjects, many days apart. Although unlikely, we tested whether the time between tasks had an influence on model-based or model-free control, rerunning both reinforcement learning models with regressors for time and its interactions with loss and risk aversion and the inverse softmax temperature from the gambling task. There was no evidence of any kind of relationship between time between tasks and either decision system. The fact that there is strong evidence of a relationship between loss aversion and model-based control even though participants performed the tasks on different days seems somewhat remarkable. However, some caution is warranted. The test-retest reliability of model-based and model-free control has not yet been established, and for loss and risk aversion the only study we are aware of measured test-retest reliability just a week apart (Glöckner and Pachur, 2012). Our findings are consistent with the idea that loss aversion and model-based control are trait variables that persist across time. However, further work is still necessary to explicitly test the stability of each measure, not only to frame the current results, but also the many other individual difference studies being conducted with each measure.

The observed result is correlational in nature. Directly manipulating each quantity would help determine whether there is a causal relationship, and if so, what the timescale and nature of the interaction is. Understanding the factors that causally influence loss aversion is a relatively unexplored area of research. One perspective comes from Stewart and colleagues in their work on decision by sampling (Stewart et al., 2006), which attempts to provide a mechanistic explanation of valuation from which loss aversion and other common properties of value functions emerge. In their model, values are constructed from a series of binary comparisons between the item in question and a sampling of similar items in memory. Loss aversion emerges, on average, because the environment is thought to contain many more small losses than smalls gains (an idea they motivate through the analysis of bank records). The theory has been used to manipulate loss aversion in laboratory experiments on short timescales (Walasek and Stewart, 2015). A hybrid experiment can be constructed, based on the same principles, that simultaneously measures model-based control. Testing the other direction, model-based control can be disrupted with transcranial magnetic stimulation (Smittenaar et al., 2013).

Finally, it should be noted that we used the most common version of the two-step task where rewards are entirely in the gain domain (Daw et al., 2011; Otto et al., 2013, 2014). A relationship between model-based control and loss aversion in a gain only version of the task suggests that the link between the two is insensitive to the signs of the outcomes in the model-based task. A second possibility, not mutually exclusive from the first, is that participants represent “no reward” as a loss. Replacing 0 and 1 outcomes by −1 and 1, setting the reference point to 0 instead of 0.5, and multiplying the three inverse softmax temperature parameters by 0.5 in the hybrid reinforcement learning model of the task results in the same data likelihood (see section Methods). It is not possible to tell with the current data which representation is being used, which remains a question for future work.

## Data Availability

The datasets for this study are available on request to the corresponding author.

## Ethics Statement

This study was carried out in accordance with the recommendations of the Institutional Review Board at Virginia Tech with written informed consent from all subjects. All subjects gave written informed consent in accordance with the Declaration of Helsinki. The protocol was approved by the Institutional Review Board at Virginia Tech.

## Author Contributions

PM and TL designed the Roanoke Brain Study. AS performed the analyses in this paper. AS, TL, and PR wrote the manuscript.

### Conflict of Interest Statement

The authors declare that the research was conducted in the absence of any commercial or financial relationships that could be construed as a potential conflict of interest.
