# Supplementary material for: Loss Aversion Correlates With the Propensity to Deploy Model-Based Control
Source: Front Neurosci. 2019 Sep 6;13:915. doi: 10.3389/fnins.2019.00915 (PMC6743018; doi:10.3389/fnins.2019.00915)
Supplement: Supplementary file 1 [file Data_Sheet_1.PDF]

Supplementary Tables and Figures

Table S1

Group level estimates for auxiliary parameters from the logistic regression / prospect theory analysis.

Parameter & Description	Median & 95% credible interval
Intercept	1.368 (1.178, 1.560)
common transition main effect	-0.034 (-0.076, 0.009)
loss main effect	0.123 (-0.084, 0.332)
risk main effect	-0.018 (-0.241, 0.204)
gamble inverse temperature main effect	0.333 (0.007, 0.660)
loss × common transition	-0.030 (-0.075, 0.015)
risk × common transition	-0.007 (-0.053, 0.039)
gamble inverse temperature × common transition	0.014 (-0.051, 0.078)
κ<br>Loss aversion weight, exp-transformed from log scale	1.249 (1.143, 1.367)
γ_{mixed}<br>Risk preference for mixed valence trials	0.815 (0.748, 0.886)
γ_{gain}<br>Risk preference for gain only trials	0.827 (0.778, 0.873)
θ<br>Gamble task inverse temperature	2.067 (1.771, 2.402)

Table S2

Group level estimates for auxiliary parameters from the hybrid reinforcement learning model / prospect theory analysis.

Parameter	Description	Median & 95% credible interval
α	Decay (learning) rate, logistic scale	0.086 (0.062, 0.114)
p	Perseveration	0.798 (0.672, 0.929)
β_2	Second-stage inverse temperature	12.508 (10.974, 14.145)
κ	Loss aversion weight, exp-transformed from log scale	1.250 (1.142, 1.368)
γ_{mixed}	Risk preference for mixed valence trials	0.815 (0.748, 0.886)
γ_{gain}	Risk preference for gain only trials	0.827 (0.779, 0.873)
θ	Gamble task inverse temperature	2.088 (1.794, 2.420)

Table S3

Group level estimates for auxiliary parameters from the logistic regression / mean–variance analysis.

Parameter & Description	Median & 95% credible interval
Intercept	1.369 (1.174, 1.564)
common transition main effect	-0.036 (-0.078, 0.006)
loss main effect	0.140 (-0.074, 0.357)
risk main effect	0.072 (-0.167, 0.313)
gamble inverse temperature main effect	0.224 (-0.023, 0.468)
loss × common transition	-0.024 (-0.070, 0.021)
risk × common transition	-0.011 (-0.061, 0.040)
gamble inverse temperature × common transition	0.002 (-0.050, 0.055)
κ<br>Loss aversion weight, exp-transformed from log scale	1.007 (0.898, 1.129)
w<br>Risk preference	0.139 (0.115, 0.164)
θ<br>Gamble task inverse temperature	1.326 (1.165, 1.506)

Table S4

Group level estimates for auxiliary parameters from the hybrid reinforcement learning model / mean–variance analysis.

Parameter	Description	Median & 95% credible interval
α	Decay (learning) rate, logistic scale	0.086 (0.063, 0.115)
p	Perseveration	0.799 (0.673, 0.928)
β_2	Second-stage inverse temperature	12.512 (10.969, 14.129)
κ	Loss aversion weight, exp-transformed from log scale	1.006 (0.895, 1.128)
w	Risk preference	0.139 (0.115, 0.164)
θ	Gamble task inverse temperature	1.327 (1.163, 1.511)

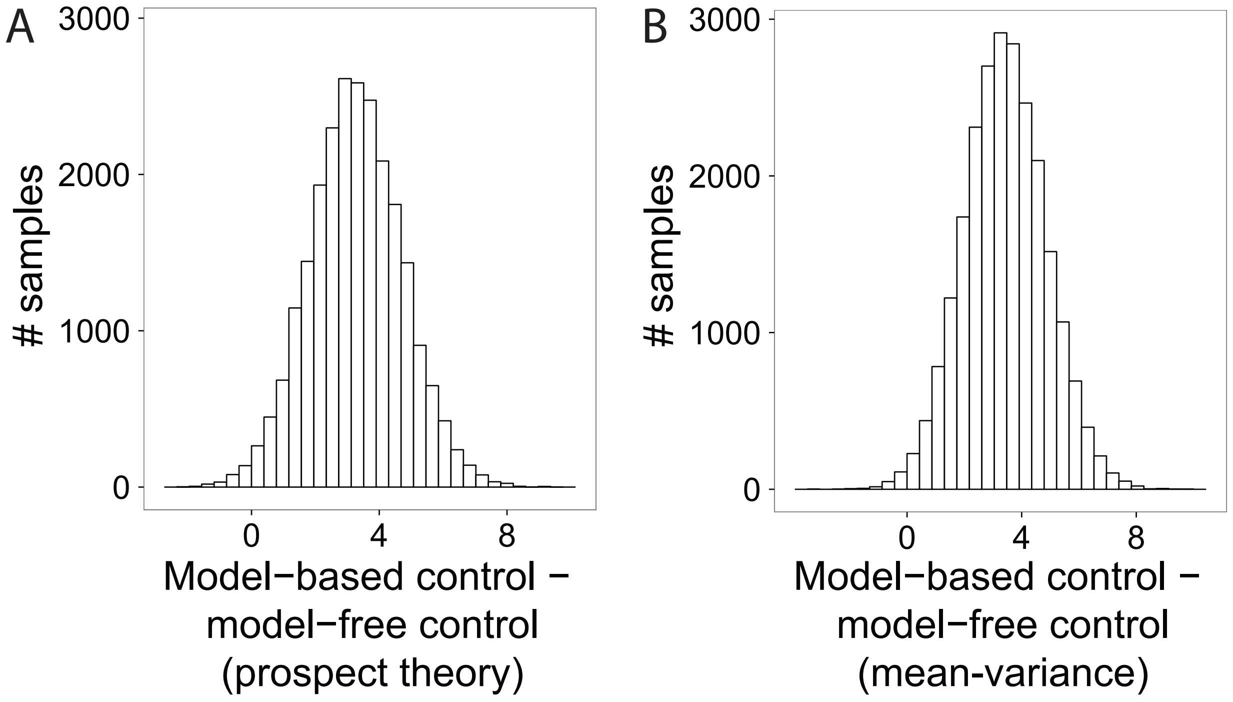

Figure S1. Distribution of the difference between the effect of loss aversion on model-based ($\beta_{mb,loss}$) and model-free ($\beta_{mf,loss}$) control, estimated using the hybrid reinforcement learning model for the two-step data and two different utility functions for the gambling task data. See also Fig. 2 and Tables 2 and 4 in the main text.

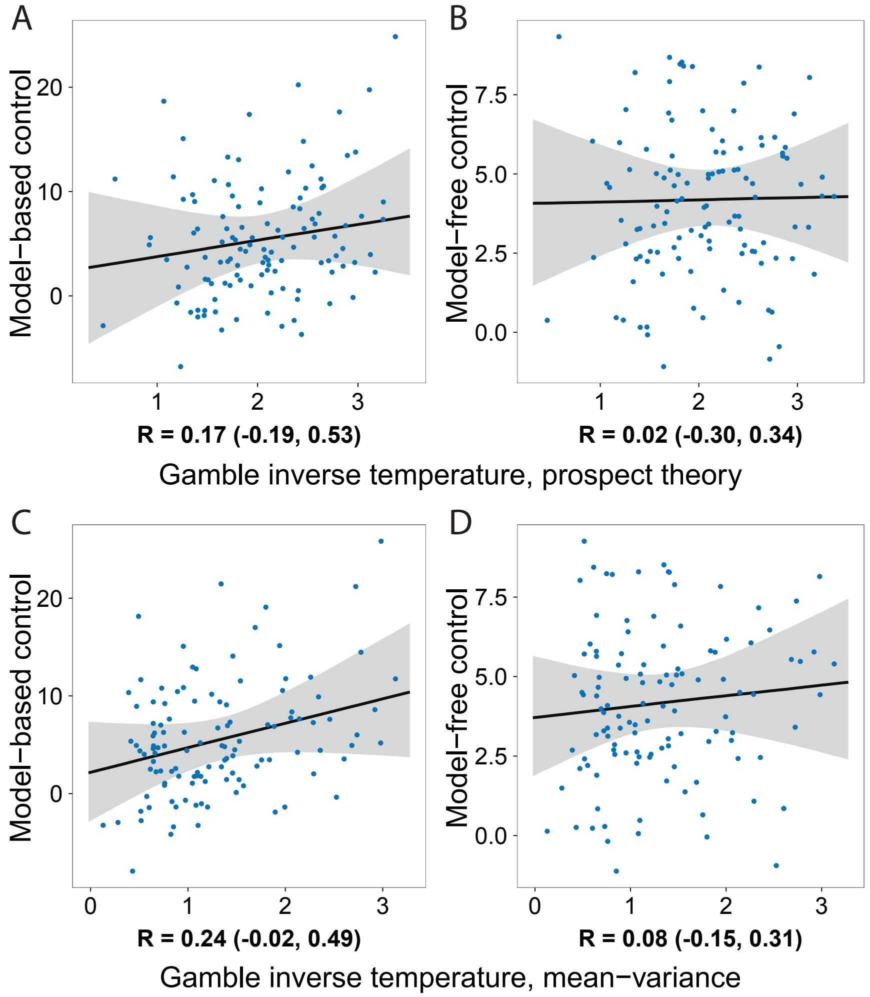

Figure S2. The degree to which individuals deploy each decision system plotted against their gambling task inverse softmax temperature, after removing the effects of loss and risk aversion. No significant relationship was observed with either decision system.
